# The State-of-the-Art Sensing Techniques in Human Activity Recognition: A Survey

**DOI:** 10.3390/s22124596

**Published:** 2022-06-17

**Authors:** Sizhen Bian, Mengxi Liu, Bo Zhou, Paul Lukowicz

**Affiliations:** German Research Centre for Artificial Intelligence (DFKI), 67663 Kaiserslautern, Germany; mengxi.liu@dfki.de (M.L.); bo.zhou@dfki.de (B.Z.); paul.lukowicz@dfki.de (P.L.)

**Keywords:** human activity recognition, sensing technique

## Abstract

Human activity recognition (HAR) has become an intensive research topic in the past decade because of the pervasive user scenarios and the overwhelming development of advanced algorithms and novel sensing approaches. Previous HAR-related sensing surveys were primarily focused on either a specific branch such as wearable sensing and video-based sensing or a full-stack presentation of both sensing and data processing techniques, resulting in weak focus on HAR-related sensing techniques. This work tries to present a thorough, in-depth survey on the state-of-the-art sensing modalities in HAR tasks to supply a solid understanding of the variant sensing principles for younger researchers of the community. First, we categorized the HAR-related sensing modalities into five classes: mechanical kinematic sensing, field-based sensing, wave-based sensing, physiological sensing, and hybrid/others. Specific sensing modalities are then presented in each category, and a thorough description of the sensing tricks and the latest related works were given. We also discussed the strengths and weaknesses of each modality across the categorization so that newcomers could have a better overview of the characteristics of each sensing modality for HAR tasks and choose the proper approaches for their specific application. Finally, we summarized the presented sensing techniques with a comparison concerning selected performance metrics and proposed a few outlooks on the future sensing techniques used for HAR tasks.

## 1. Introduction

A better understanding of human behavior benefits individuals on a large scale, including healthcare, well-being, social interaction, life assistance, etc. Thus human activity recognition (HAR) has been tremendously explored in recent years, driven by the enormous technical advances in sensing, computation, and immense human-centric user scenarios. The explosive advancement in machine learning and hardware architecture has dramatically improved the accuracy and robustness of HAR tasks and enabled the technique to be deployed at the far edge near the body. Besides the computational ability, the sensing technique plays a fundamental and critical role in HAR tasks. Therefore, a broader range of sensing modalities has been explored in recent years, aiming to boost the development of reliable body activity digitalized recording. The proposed sensing modalities range from traditional motion sensing methods such as accelerometers, to novel TOF-based sensing such as mmWave, from neural network-aided image processing for activity abstraction to very straightforward proximity detection approaches such as RF-tags.

**Figure 1:**
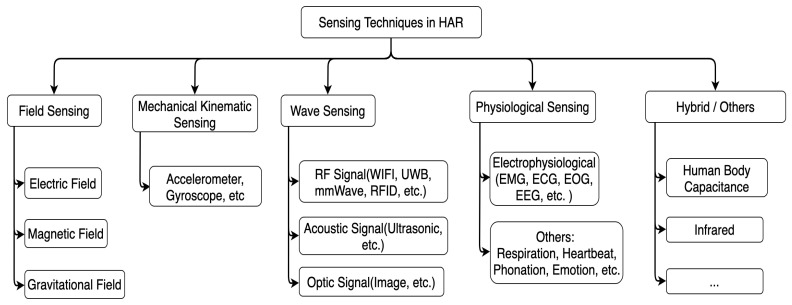
Sensing techniques in human activity recognition.

To acquire a comprehensive overview of the state-of-the-art sensing modalities in human activity recognition, categorization of the adopted sensors is an efficient approach for a deeper understanding of the sensing medium. Researchers have already categorized related sensors into different classes, such as active and passive sensors depending on the need for external excitation [1], or intrusive and non-intrusive sensors depending on the interference of the sensors in the process flow [2,3]. With a further step, we elaborately categorized the HAR-related sensing modalities into five classes depending on the following sensing principles: kinematic sensing, field-based sensing, wave-based sensing, physiological sensing, and hybrid or other approaches, as Figure 1 presents. We enumerated most of the sensing modalities within each class with an in-depth description of the sensing tricks in the HAR tasks.

### 1.1. Relevant Surveys

Despite the enormous scope of sensing modalities in HAR tasks, related survey works are limited. The existing surveys on HAR sensing are primarily focused either on a specific scenario (such as wearable sensing or video-based sensing) or on full-stack presentation of both sensing and data processing techniques, which results in a weak focus on HAR-related sensing techniques. Table 1 lists the latest HAR sensing-related surveys in recent years from the literature. Those high-related surveys (as well as other references listed in this paper) are first searched using keywords such as human activity recognition, survey, overview, and sensing technique, from platforms including Google Scholar, IEEE Xplore, Microsoft Academic, etc. Second, the survey papers cited in the searched surveys were also considered. As can be seen, nearly all the exiting surveys only focused on a specific domain of HAR sensing techniques, such as device-free sensors [4], smartphone sensors [5], radar sensors [6], etc. Such surveys could supply a detailed research result on the particular sensing domain but lack focus on the adopted sensors in HAR. In contrast, there are only a few surveys [7,8] that supply thorough sensor modalities. However, an in-depth introduction and comparison of the sensing tricks is still lacking.

### 1.2. Paper Aims and Contribution

This work tries to fill the gap by presenting an extensive and in-depth survey on the state-of-the-art sensing modalities in HAR tasks, aiming to supply a solid understanding of most sensing modalities for researchers in the community. Overall, we provide the following contributions in this survey:

1. For a clear overview of the multifaceted nature of HAR tasks, we firstly sorted the human activities into three types: body position-related services (“where”), body action-related services (“what”), and body status-related services (“how”). Such sorting coarsely but briefly introduces the final objective of the utilized sensing technique, which supplies the readers with an elementary step for the sensing concept.
2. We then categorized the sensing techniques in HAR tasks into five classes based on the underlying physical principle: mechanical kinematic sensing, wave-based sensing, field-based sensing, physiological sensing, and hybrid or others. We enumerated broadly the adopted sensing modalities within each category and supplied an in-depth description of the underlying technical tricks. Such a sensor-oriented categorization supplies the readers a further understanding of the distinct HAR tasks.
3. We gave each sensing modality an in/cross-class comparison with eight metrics to better understand each modality’s limitation and dominant properties and its typical applications in HAR. Finally, we provided a few insights regarding its future development.

This survey is constructed as follows: in Section 1, we briefly stated the motivation of this survey considering the existing works and the development of the state-of-the-art HAR sensing techniques. We then summarized and categorized all human activities in the research scope according to the activity attribute in Section 2, followed by a brief description of the general process of the HAR task. Section 3 showed our categorization of the current HAR sensing techniques and gave an in-depth and extensive description of each sensing modality, followed by a summary regarding eight critical sensing performance metrics. Section 4 and Section 5 presented a few outlooks into the future development of the HAR-related sensing techniques and the conclusion of our work.

## 2. Background

### 2.1. Object of Human Activities Recognition (HAR)

**Figure 2:**
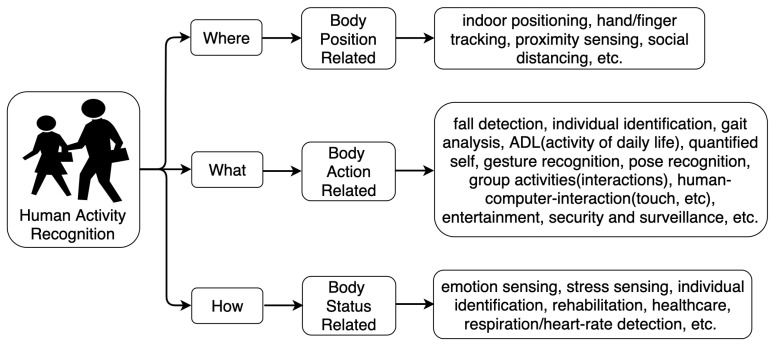
Categorization of human activities.

Human activities refer to human behaviors concerning the body or the environment. The recognition of human activity aims to capture the action/status of the agents from a series of observations. A successful recognition could provide personalized support in plenty of human-centric applications [16,17]. Since the HAR tasks cover a wide range of activities, it is necessary to sort the related topics in an impressive and compact way. Most research works assort the task into a few levels according to the activity complexity [4,7] (from gestures to actions), followed by human object/human interaction. Group activities [18,19] are the most complicated ones, requiring multiple people and essentially composed of series of gestures, actions, and interactions. In this work, we sorted the human activity recognition into three problems (Figure 2) according to the attributes of the targeted task: body position-related problem, body action-related problem, and body status-related problem corresponding to the questions of “where”, “what”, and “how”, respectively. The“where” problem addresses the position-related recognition, such as indoor positioning [20], tracking [21], proximity [22], etc. The “what” problem deals with the action-related recognition, which belongs to the most widely researched section under the HAR task. Examples are fall detection [23], gait analysis [24], ADL (activity of daily life) [25], etc. The last one is the “how” problem, inferring the body status-related research, such as emotion-sensing [26], respiration/heartrate sensing [27], healthcare [28], etc. This task-oriented categorization aims to supply a basic concept of the objectives of human activity recognition. As can be seen, HAR is a multifaceted topic covering almost all human-related activities and needs interdisciplinary knowledge to understand the behaviors and provide assistance properly.

### 2.2. General Process of Human Activity Recognition

**Figure 3:**
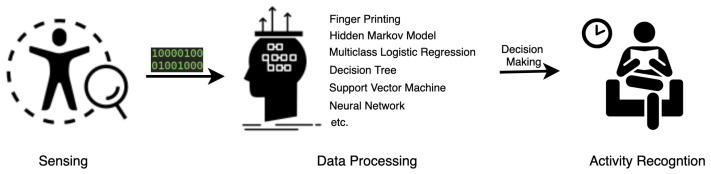
The general process of an HAR task.

Human activity recognition explains comprehensive body behaviors aiming to supply ethical-respect assistance. A complete recognition task is generally composed of three steps (Figure 3): sensing, data processing, and decision making. Sensing techniques play a fundamental role in the procedure, trying to perceive as much contextual knowledge as possible so that a reliable recognition becomes possible. A successful HAR task depends firstly on the data quality perceived from the applied sensors and secondly on the processing skills of the acquired data. With the developments in physics, electronics, and other fundamental subjects, novel sensors and devices are emerging to supply more efficient signal patterns for human activity recognition [29,30,31]. The revolution of the ToF camera, as an example, has enabled the camera to move from simply capturing the streamed images to providing additional depth information to the images, thus provoking a wide range of recognition tasks such as hand gestures [32] and facial expressions [33]. Recently, significant advances in detection accuracy and range, and the power consumption of the ToF sensor, have continued to boost novel applications in both industrial automation [34] and consumer electronics [35]. Diverse sensing techniques have been utilized for specific HAR scenarios and have provided outperforming recognition performance, which motivated us to write this survey focusing on those state-of-the-art sensing techniques with in-depth exploration and extensive analysis. After getting the knowledge from the sensing approaches, the second step is to process the data. According to the data quality and the deployed algorithms, a pre-processing step such as normalization or calibration might be needed. For small volume data such as single-dimensional ECG data or RSSI-based positioning data, a rule-based [36] or multi-lateration [37] algorithm will supply good results. For extensive volume data such as image and speech, the algorithms deployed on the pre-processed data have been dominated by deep neural network-based models [38] with a training process due to its cutting-edge recognition performance compared with traditional approaches [39], such as feature descriptors in object detection. Currently, a large amount of HAR tasks are conducted based on the image streams captured from different kinds of cameras. Those works were focused on spatio-temporal relations of the individuals in the scene. Traditionally, researchers handcraft the features [40] to deduce the target activity, but such approaches firstly heavily rely on the individual experience for the selection of high-relative features; secondly, the handcrafted features might be inefficient and lack generalization in dynamic and environments. The last decade’s exploration of machine learning has impressively influenced the processing pipeline in HAR applications. Network models based on convolutional computing [41] or attentional mechanisms [42] for feature abstraction have dominated the approaches for data processing and presented the state-of-the-art recognition performance. The corresponding general framework comprises steps including data acquisition from the applied sensing technique, feature abstraction with distinct network models, and target decision-making based on the inference result of the network model [43,44]. After the patterns of the activities are acquired from the data in the processing step, a decision on the activity recognition could be concluded as a final step. This survey will, however, not cover the recognition algorithms adopted in the data processing step and the final inference step based on the network models. The aim of this survey is to supply a detailed explanation of the physical principles under the applied sensing techniques in HAR tasks and discuss the differences between them so that researchers can choose the right one for their applications. As the first component in the pipeline of the HAR application, the sensing techniques transform human physical activities into numerical information that could be further processed. The following section will extensively present the related HAR-targeted sensing approaches and the behind.

## 3. Sensing Techniques

As Figure 1 depicts, we categorized the sensing techniques into five classes according to the sensing principles: mechanical kinematic sensing, field sensing, wave sensing, physiological sensing, and the hybrid or others. Compared with other categorization approaches such as the deployment approach (wearable, object, environmental, etc.), the principle-based categorization gives a better understanding of the sensing technique’s physical background. In the following subsections, we will enumerate the leading sensing modalities in each class with their sensing tricks and related state-of-the-art research works. After the enumeration, we also provide an evaluation and comparison of the sensing modalities with the following performance metrics:

- Cost. Low: less than 10 USD. High: hundreds to thousands of USD.
- Power efficiency. Low: level of mW. High: level of W.
- Signal source. Active or passive according to the source of the measured physical characteristics (naturally or emitted by the sensing system).
- Robustness. The ability to tolerate perturbations that might affect the performance of the sensor.
- Privacy concern. If the sensing approach records individual information beyond the need of interest.
- Computational load. The demand of the hardware resources for successful decision making.
- Typical application. A list of HAR tasks being addressed by the sensing approach.
- Other criterion. Such as installing/maintaining complexity, environmental dependency (line of sight, etc.), accuracy and sensitivity, etc.

### 3.1. Mechanical Kinematic Sensing

Mechanical sensing refers to mechanical mobility and deformation when a force is deployed on/from the target. The mobility and deformation are perceived by the mechanical sensors, which transform the mechanical variation into electric signals. Mechanical sensors have been widely used to monitor body activity such as the kinematic senors.

In physics and maths, kinematics is a field of study exploring geometrical motion. Kinematics sensing in HAR is based on the human body-related motion properties such as velocity, acceleration, rotation, etc. Since the recognition of body motion activities is the most related object of HAR tasks compared to other objects such as positioning, status monitoring, etc., kinematic sensors have become the dominant sensing approach in scientific research and industry application. The most popular deployed sensors are inertial ones such as accelerometers and gyroscopes. Another reason for the massive usage of inertial sensors is the power effectiveness and small size, enabling a pervasive embedding of the sensing unit into personal assistant devices such as smartphones and wearable devices such as fitness bands.

Nearly all of the current commercial wearable devices are embedded with inertial sensors that deliver motion signals of a distinct body part without much concern about power consumption and comfort. Both academic and industrial researchers have developed plenty of works with inertial-sensor-embedded wearable applications. For example, Hristijan et al. [45] explored a weighted ensemble learning algorithm with data from head-mounted inertial sensors to recognize eight everyday activities. Tobias et al. [46] proposed a respiration rate monitoring using an in-ear headphone inertial sensor. Wrist-, hand-, finger-worn inertial sensors are primarily used for gesture recognition as a means of human–machine interface [47,48,49]. Related wearables are smart gloves, smart watches, smart rings, wristbands, etc. Another popular motion-recognition-enabled wearable modality is the smart garment. Kang et al. [50] designed an IMU and conductive-yarn-integrated clothes to prevent spinal disease by continuous posture monitoring. Zhang [51] evaluated an innovative full-body wearable garment system based on IMUs for motion analysis during different exercises. Wang et al. [52] evaluated stroke patients’ acceptance of an IMU-embedded smart garment for supporting upper extremity rehabilitation and received positive responses in a clinical setting. Besides the wearable electric devices and smart garments, inertial sensors could also be integrated into shoes and soles for foot- and leg-related motion-based research, such as gait analysis [53], indoor pedestrian navigation [54], workout recognition [55], injury prevention [56], etc.

Besides the advantage in wearability (power consumption, small size, low cost, pervasiveness), inertial sensors also outperform in data quality regarding sensitivity and accuracy. A high-resolution accelerometer could sense minor vibrations on bodies. Cesareo et al. [57] assessed breathing parameters using the IMU-based system. With the proposed algorithm, they reconstructed respiration-induced movement and precisely perceived the respiratory rate through an automatic method. Huang et al. [58] demonstrated a novel method for 3D pose reconstruction with six IMUs, which outperformed the camera-based methods in situations such as heavy occlusions and fast motion.

Regarding the above-listed advantages, inertial sensors currently play the most critical role in HAR tasks, even in the unique cases of commercial wearable targeting motion-related applications [59]. However, inertial sensors need to be mounted on the target part to sense the part motion pattern, which might be annoying regarding the user habit when long-term continuous motion monitoring is demanded and might cause burden and discomfort for users. For highly accurate motion reconstruction, the inertial sensor also faces the challenge of accumulated errors, which need to addressed by constant recalibration.

### 3.2. Wave Sensing

Wave sensing is a non-contact sensing technique based on the propagation properties of waves. Three kinds of wave sensing approaches are mainly used for HAR tasks. The first is the RF signals such as WiFi, BT, mmWave, etc., referring to a wireless electromagnetic signal with identified radio frequencies ranging from 3 kHz to 300 GHz. The propagation of the wireless electromagnetic wave is based on the electric and magnetic fields that are orthogonal to each other. The second wave signal is the acoustic signal, a mechanical wave that includes vibration, sound, ultrasound, and infrasound. The third is the optical signal, an electromagnetic signal with the typical extremely high frequency in THz order. In HAR, those wave sensing approaches have been explored widely and deeply. For example, image-based activity recognition analyzes the target actions in the images from the video and can supply recognition with high accuracy. Since video information is captured by a camera that takes all light rays and focuses it via the lens onto a grid of tiny light-sensitive photosites, it is essentially optic-enabled sensing. RF and acoustic signals, as ambient sensors, offer advantages in both privacy protection and reducing the extra burden of objects.

**Figure 4:**
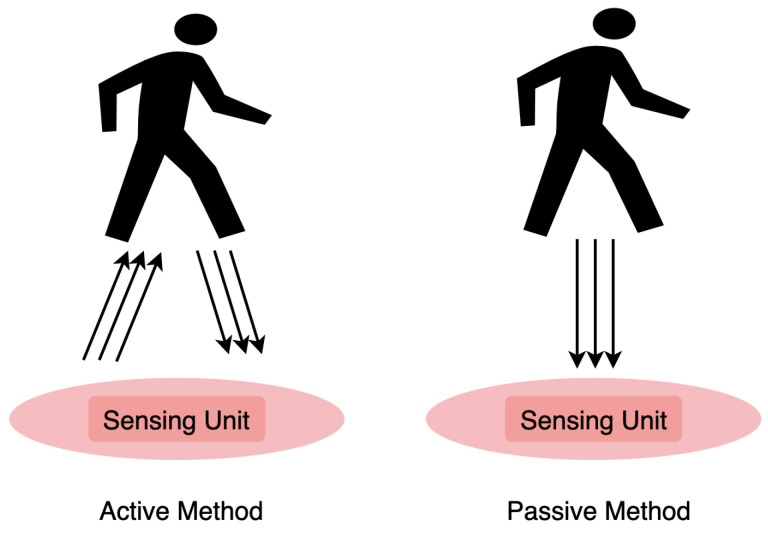
Wave-based human-centric sensing in two methods: active and passive.

Two kinds of sensing methods exist in wave-based human-centric sensing: active and passive sensing. Figure 4 shows the essential difference between the two methods. Active sensing requires an external source of energy. The source emits waves to the measured object and receives the wave’s reflection, transmission, and absorption. Features abstracted from the received information are then utilized for object description. On the other hand, passive sensing does not need an active wave source and perceives the object variables by receiving a measured wave signal from the object.

(A) RF Signal

RF-based HAR is a non-intrusive approach that can bypass the burden and discomfort caused by wearable activity monitoring sensors. The basic principle of the RF-based HAR system is that the propagation path of the RF wave will be affected by the intrusiveness of the human body. The resulting variations in the received wave can then be used as features to deduce different activities.

A series of RF signals were explored for HAR tasks, such as WiFi, UWB, mmWave, etc. Among them, WiFi is the most popular due to its pervasiveness in the indoor environment. The critical intuition of WiFi-based HAR is that motions of the human body introduce different multipath distortions in WiFi signals and generate different patterns in the time series of channel state information. Li et al. [60] proposed a system named Wi-Motion, being able to jointly leverage the amplitude and phase information extracted from the channel state information sequence, and to achieve a mean accuracy of 96.6% in the line-of-sight environment and 92% in not line-of-sight environment regarding five predefined typical human activities (bend, half squat, step, stretch leg, and jump). Liu et al. [61] designed a WiFi-based sleep monitoring system to abstract fine-grained sleep information such as a person’s respiration, sleeping postures and rollovers by continuously collecting the fine-grained wireless channel state information. Besides the activity recognition, the WiFi signal can be leveraged for indoor location tasks. An example work is from Wang et al. [62] where the authors proposed a dual-task residual convolutional neural network with one-dimensional convolutional layers for the joint task of activity recognition and indoor localization. Bluetooth technology is another RF approach to perform HAR tasks. However, compared with the WiFi signal, the Bluetooth signal is relatively weak [63]. Thus the accuracy and reaching range is limited. However, it enjoys advantages in cost and ease of use. Therefore, Bluetooth technology is mainly used for indoor locations by deploying plenty of small form-factor, power-saving, cost-efficient tags with high density [64].

Besides the WiFi and BT wave signal, the mmWave technology, which operates in the frequency range of 30 GHz and 300 GHz, recently exhibited high attraction to researchers. Since a higher frequency means a smaller antenna size, thus the mmWave radar is compact in form factor. Many antennas could be packaged into a small space to enable highly directional beams. Moreover, the mmWave signal enjoys a larger bandwidth than WiFi signals and higher range resolution. Recent advances in small and low-cost single-chip consumer radar systems operating at mmWave frequencies have opened up many new applications, such as automotive radar, health monitoring, etc. HAR has also been explored with mmWave-based approaches and has received outstanding results with fine-grained classifiers. Zhang et al. [65] predicted the target behavior by using the micro-Doppler effect (induced by micromotion dynamics of a target or its structure) from mmWave radar [65]. Using a neural network work-based classifier, they got 95.19% accuracy of bulk motion of the body and the micromotions from arms and legs. Zhao et al. [66] proposed a system named mBeats, where a robot mounted with mmWave radar system is used to provide periodic heart rate measurements under different user poses. A fall-detection system based on mmWave radar was also presented by Sun et al. [67] with the support of a recurrent neural network with long short-term memory units. Li et al. [68] designed another interesting mmWave radar-enabled system called ThuMouse, which regressively tracks the position of a finger aided by a deep neural network. MmWave-related exploration is still at an early stage and will have an explosive growth period in the following years triggered by its unusual behavior compared to WiFi, BT, and the large-scale chip-level commercialization.

**Figure 5:**
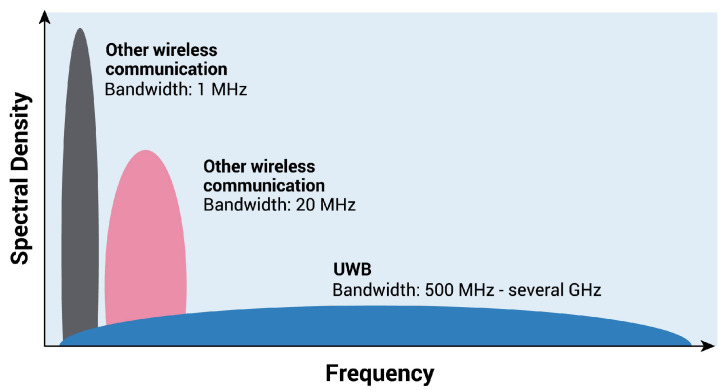
The wide UWB power spectrum results in a low power consumption compared to other technologies. (Source: FiRa Consortium).

Another greatly promising and widely used RF wave signal is the ultra wide band (UWB), which is a decades-old wireless technology used for short-range, high-bandwidth communication with a high data rate. Now it is also as a standard for high-accuracy location services. According to FiRa, a consortium founded by the dominating companies for UWB standards, the reborn UWB will mainly be focused on three use cases: hands-free access control, location-based services, and peer-to-peer communication, which will be complementary to current dominant wireless solutions. Recently, UWB support has started to appear in high-end smartphones. There is no question that the UWB will boost another wave on related applications. Figure 5 shows the wide spectrum of UWB compared with others, allowing UWB to operate at a shallow power state and build stable connectivity with other devices in a crowded radio environment. Thanks to the higher base frequency, UWB devices can provide higher accuracy in position with the level of around 10 cm [69], which is highly dominant compared with WiFi or BT-based positioning with accuracy of meter-level [70]. Another key feature is that UWB is resistive to the multipath effect, a common issue for most RF-based wave sensing technology. The multipath effect refers to the received radio signal from more than one path because of the reflection of retraction caused by objects near the main signal path. The large bandwidth of UWB provides frequency diversity that can make the time-modulated ultra-wideband (TM-UWB) signal resistant to the multipath effects [71]. Researchers have explored plentiful HAR-related applications with UWB, such as activity recognition in smart homes [72], gesture recognition [73], sleep postural transition recognition [74], healthcare monitoring [75], etc. With the popularization of low-cost UWB chips in wearable devices, there will be more short distance-based novel applications based on the UWB technique, such as swarm intelligence, social distancing, etc. However, despite the above-described advantages of UWB, there will still be some time for a wide deployment of UWB, considering its higher cost. Moreover, regarding the data streaming rate, UWB is not a good option for large data interaction between devices compared with other narrowband radio systems.

(B) Acoustic Signal

An acoustic signal is a mechanical wave resulting from an oscillation of pressure and travels through the solid, liquid, or gas in the form of a wave. A clear, well-known acoustic signal is the audible sound from a speaker by the vibration of vocal folds. The vibration travels through air and reaches the outer ear and the eardrum. There are two kinds of sound outside the range of audible sound frequency (20–20 Khz): infrasound and ultrasound. An example of infrasound is the atmospheric infrasound caused by the earthquake when the earth’s surface near the epicenter and surrounding regions oscillates in a low frequency. Ultrasound is an acoustic signal with a higher frequency than the upper audible limit of human hearings. A widely used example of ultrasound is medical imaging, where the ultrasound waves travel through the body and create a sonogram of organs, tissues, etc.

As an ambient sensor, ultrasound could firstly supply mm level positioning accuracy indoors based on the time of flight [76,77]. Such a positioning system is based on several wireless ultrasonic beacons with fixed and known coordination under an indoor environment, and receives or emits ultrasonic signals which are finally used for position deduction. The wireless module (WiFi, Bluetooth, or others) is used for data interaction and time synchronization. Finger motion recognition is another application based on ultrasound by leveraging the characteristic of detected morphological changes of deep muscles and tendons. Yang et al. [78] had obtained an accuracy of 95.4% for real-time finger motion recognition. Mokhtari et al. [79] proposed a resident identification system as an innovative home platform by using ultrasound arrays to detect the height of the moving resident and other sensors such as pyroelectric infrared to detect the moving direction. Wang et al. proposed a novel contactless respiration monitoring approach using ultrasound signals with off-the-shelf audio devices. Unlike other works based on chest displacement where false detection may often occur, they monitor the respiration by directly sensing the exhaled airflow from breathing. The principle is that the exhaled airflow from breathing can be regarded as air turbulence, scattering the sound wave and resulting in the doppler effect. The experiment’s results showed an accuracy of 0.3 breaths/min (2%), and it was concluded that the ambient noise and the variation of respiration rate, respiration style, sensing distance, and transmitted signal frequency have little effect on respiration monitoring accuracy of the system.

Previous works on sound (captured by the microphone on a smartphone) are mainly focused on the following application cases: environment assessment [80,81], proximity sensing [82,83], or indoor positioning [84,85]. The sources of sound are either from fine-tuned tags or from the surroundings. In the work of Benjamin et al. [82], an algorithm using inaudible sound patterns was explored to accurately detect whether two mobile phones are within a few meters from each other. The method can be implemented as a standard smartphone application with real-time inferencing, enabling smartphone-based collaborative activity detection and other embedded sensors.

Overall, acoustic signals provide an alternative and competitive approach for highly accurate human or robot positioning and distance-related activity recognition. The method is non-intrusive, thus reducing users’ extra burden and protecting privacy security. However, it still suffers from the computational load and is limited by complex environmental acoustic sources. For example, the accuracy and robustness of ultrasound-based indoor positioning enormously decrease when a collision-like sound occurs, or when a significant barrier between tags exists.

(C) Optic Signal

Optical signals for HAR tasks mainly refer to deep learning-enabled image processing with the images captured by the photosensitive elements in cameras. Most related works focused on spatio-temporal relations among the objects in the scene. Those works involved tracking multi-agents spots, evaluating their appearance, aggregating independent and joint features, segmenting their movements, extracting their actions, and then perceiving their activities. Image-based systems could cover almost every HAR task and achieve very high recognition accuracy because of the complete view of data captured in the scene. The covered tasks include positioning, navigation, body-part monitoring, full-body monitoring, individual activity recognition, group activity recognition, etc. Sathyamoorthy et al. [86] designed a system named COVID-robot for social distancing monitoring in crowded scenarios. With the help of an RGB-D camera and a 2-D Lidar, the mobile robot can avoid collision in a crowd and estimate distance between all detected individuals among the camera view during self-navigating. Lee et al. [87] presented a innovative wearable navigation system based on an RGBD camera to help the visually impaired. A glass-mounted RGBD camera collected the environment information, which is as a input to their navigation algorithm of real-time 6-DOF feature-based visual odometry. Kim et al. [88] proposed a hand gesture control system based on the tactile feedback to the user’s hand. Amit et al. [89] proposed an approach to analyze a user’s body posture during a workout and compare it to a professional’s reference workout, thus getting visual feedback while performing a workout. The system aims to assist people in completing the exercises independently and prevent incorrectly performed motions that may eventually cause severe long-term injuries. Meng et al. [90] addressed the problem of recognizing person–person interaction by depth cameras providing multi-view data. They divided each person–person interaction into body part interactions at first. Then the pairwise features of these body part interactions were used to analyze the person–person interaction. The method was demonstrated in three public datasets. As can be seen, the image-based HAR tasks are profoundly dependent on the neural-network-based algorithms. Most of the researcher’s effort in this field is in the advanced algorithm exploration to reach the state of the art.

Undoubtedly, camera-based HAR systems have succeeded in different scenarios, including indoor monitoring and outdoor surveillance. However, the problem is that the approach might not be well accepted due to severe privacy concerns. This is one reason that sensor-based HAR is still prevalent in research communities and has led to many research contributions recently. Another significant disadvantage of an image-based solution is located in the computation load. Since the image-based HAR needs strong hardware support (GPU, CPU, memory, bus) for running the millions of parameters (weights and activations) from the trained deep neural network, the cost of hardware resources, power, and maintenance is enormous. Additionally, since this is an optic sensing solution, the performance is deeply influenced by environmental conditions such as light, temperature, air quality, etc.

### 3.3. Physiological Sensing

**Figure 6:**
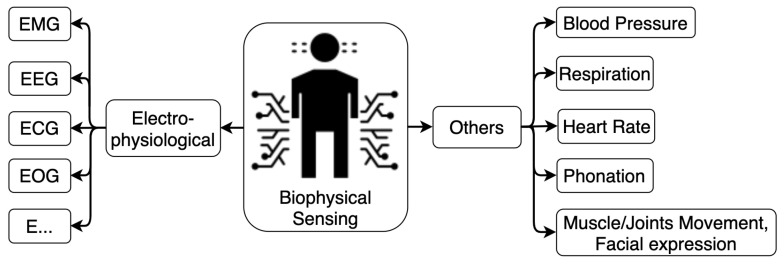
Physiological sensing modalities for HAR.

The term “physiological sensing” refers to both the natural physiological signals and the kinematic signals activated from the organism. Physiological variables have been widely used in diagnosis, drug discovery, healthcare monitoring, etc. In human activity recognition, the human body, a compound of biochemistry, has a rich set of electrophysiological and kinematic variables that could be measured on the body to indicate the status and action of the object. Figure 6 summarizes the biological variables used in the task of HAR.

(A) Electrophysiological Signals

Electrophysiology focuses on the electrical properties of the neurons, molecular and cellular, of living beings. The behavior of neurons is essentially based on the electrical and chemical signals inside the physical body. A series of high-level expressions and actions could be interpreted by monitoring those signals. EMG (electromyography), ECG (electrocardiogram), EEG (electroencephalogram), and EOG (electrooculography) are commonly monitored electrophysiological signals in clinical scenarios. Research works in the last decade showed a significant contribution of electrophysiological signals in human behavior interpretation. For example, electromyography is a diagnostic procedure that monitors the electrical signals of muscles and motor neurons. Pancholi et al. [91] developed a low-cost EMG sensing system to recognize the arm activities such as hand open/close or wrist extension/flexion. Srikanth et al. [92] focused on the recognition of complex construction activities with wearable EMG and IMU sensors in a neural network-based way. Similar work has been explored for hand gesture recognition [93,94], human–computer interaction [95,96], etc. ECG records the electrical signal during the heartbeat. With up to twelve electrodes, ECG signals are commonly used to check different heart conditions. The ECG signal is also a popular explored signal for HAR and commonly combined with other inertial sensors [97,98]. Since the cells in the brain communicate through fast electrical impulses, researchers developed EEG equipment to record the brain’s electrical activity by using small metal electrodes attached to the scalp [99]. The signal was also explored in HAR such as eyes open/close [100], emotion recognition [101], etc. EOG is a technique for recording the capitalization on the eyes’ cornea–retina potential difference. Typical basic applications of EOG signals are ophthalmological diagnosis and eye movement recording. However, researchers have already explored the potential of EOG signals in HAR [102]. Lu et al. [103] also proposed a dual model to achieve EOG-based human activity recognition with an average recognition accuracy of 88.15% according to three types of activities (i.e., reading, writing, and resting). Besides the above-listed commonly used electrophysiological signals, many other related signals describing various electrical body-related variables could be explored for HAR tasks. Electrophysiological signals need more effort for activity interpretation compared with other sensing approaches because of the complexity of body anatomy and are used mostly as an auxiliary role. However, they have advantages such as ubiquity and the on-body measurement, indicating the potential of wearables in the implementation stage.

(B) Other physiological signals

An example is from Paolo Palatini’s study [104] exploring the relation between sports and blood pressure. One of the conclusions is that both systolic and diastolic blood pressure increase significantly during weight lifting, which is a solid support to the current belief that people with hypertension should not take isometric sports. Besides the blood pressure observation, monitoring kinematic signals such as respiration and heart rate plays a critical role in sleep studies, sports training, patient monitoring, etc. Lu et al. [105] designed a wearable sensor system with the fusion of heart rate, respiration, and motion measurement sensors to enhance the energy expenditure estimation. Their study shows that the fusion design supplies more stable estimation than existing systems. Brouwer et al. [106] improved real-life emotion estimates based on heart rate. Li et al. [107] proposed a sleep and wake classification model with heart rate and respiration signals for long-term sleep studies and reached 88% classification accuracy. Plenty of research work utilized the two sensing modalities in wearable configuration to monitor medicine and health state [108,109]. Phonation is when the vocal folds produce certain sounds through vibration, which has also been explored to help disabled and unhealthy individuals for a better expression or understanding. Lee et al. [110] developed a lip-reading algorithm using optical flow and properties of articulatory phonation for hearing-impaired people, supplying them with continuous feedback on their pronunciation and phonation through lip-reading training, aiming for more effective communication with people without hearing disabilities. Gomez et al. [111] proposed a monitoring approach of Parkinson’s disease leveraging biomechanical instability of phonation for the frequent evaluation at a distance. Muscle (either on facial or other body parts) and joint movement monitoring is a more straightforward way for human activity recognition. The movement can be perceived by a series of sensors such as fabric stretch sensors, capacitive sensors, laser doppler vibrometry, etc. Applications based on muscle/joint movement monitoring include hand gesture recognition [112], physical stress [113], gait cycle estimation [114], chronic pain level recognition [115], etc. As electrophysiological sensing, kinematic biological sensing is an on-body approach that the monitoring can be placed near the body, enabling continuous observation and remote feedback, especially for healthcare, diagnosis, and rehabilitation applications.

### 3.4. Field Sensing

The field is a concept in physics, inferring a region in which each point will be affected by force. For example, electric charges will form an electric field. When another charged particle is placed in the electric field, it will bear an electric force that either repels or attracts it. A magnet will generate a magnetic field surrounding it, and a paper clip in the range of the field will be pulled towards the magnet. Two like magnetic poles will also repel each other when they are close enough to be in the range of either magnetic field. Any object with a quality on Earth will fall to the ground because of its gravity, as it is affected by the force of Earth’s gravitational field.

The field strength means the magnitude of a vector-valued field. For example, in the electric field, the strength is represented by the unit of volts per meter (V/m). In the magnetic field, the field is represented by Oersted*Ampere/meter (Oe*A/m). Moreover, when the flux density defines the strength, the Gaus (G) units or Tesla (T) are used. The gravitational field strength is measured in meters per second squared (m/s^2^) or Newtons per kilogram (N/kg). All the units used to represent the field strength are vector-valued. Another approach to know the field strength is to look at the field contour lines. The closer the lines are, the stronger the forces in that part of the field are, and the stronger the field strength is.

**Figure 7:**
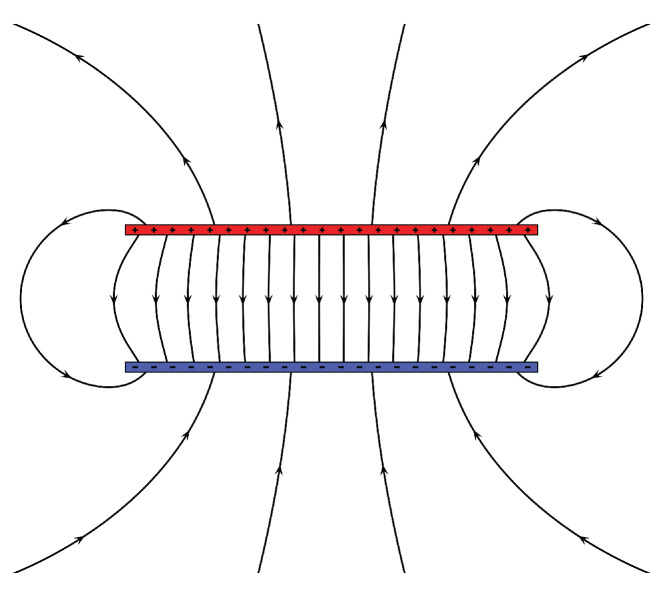
Eletric field (parallel plate capacitor).

**Figure 8:**
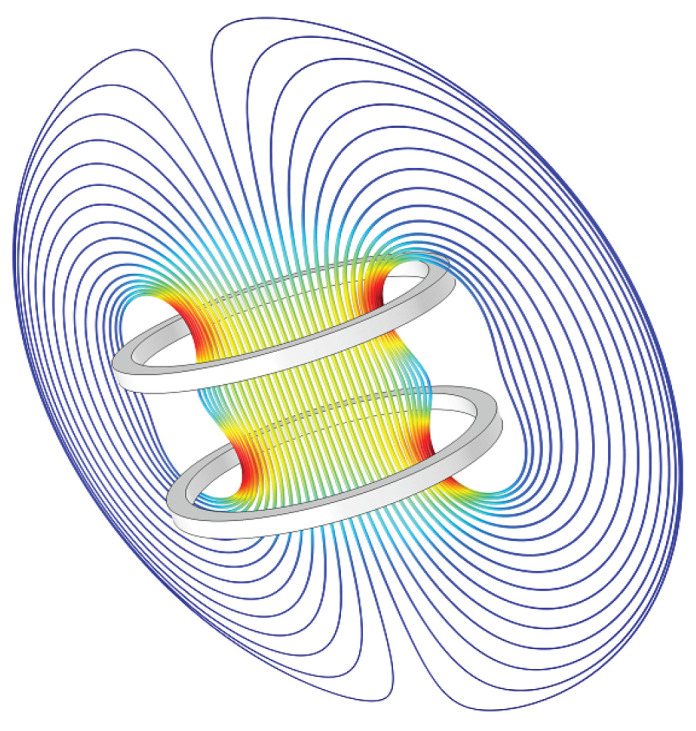
Magnetic field (Helmholtz coils).

**Figure 9:**
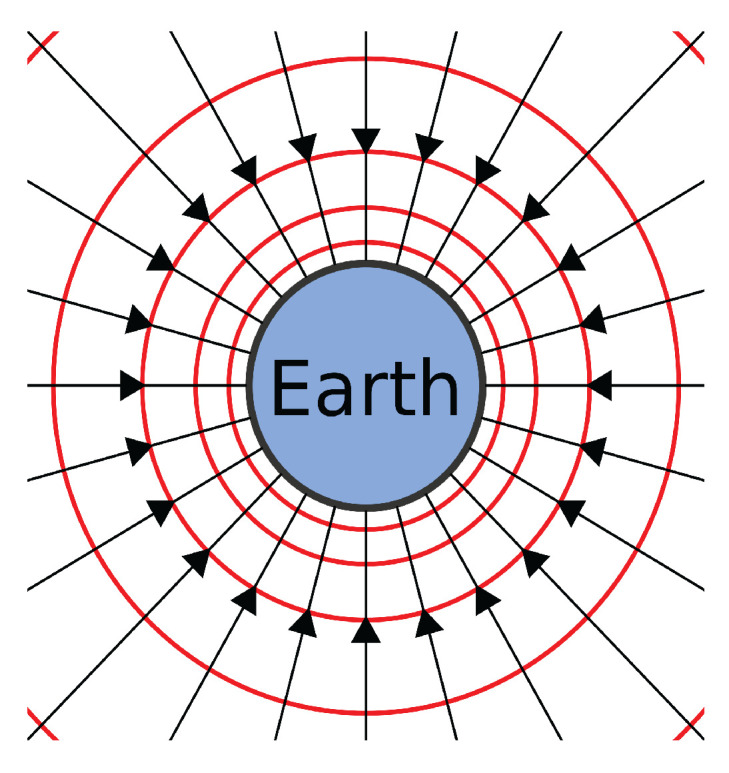
Gravitational field of Earth.

Figure 7, Figure 8 and Figure 9 show an electric field of a parallel plate capacitor, a magnetic field activated by a Helmholtz coil, and the gravitational field of the Earth, respectively. Field-based sensing is based either on the field strength measurement (such as magnetic field strength) or the strength variation caused by characteristics indirectly (such as the potential change of the capacitor, the pressure of object caused by the gravity).

(A) Electric Field

The electric field is ubiquitous in our environment since any potential difference will construct an electric field. Either powered objects (such as appliances, walled power cables, etc.) or non-powered conductive items (such as metal frames near the power cable in a building, the human body, etc.) will activate an electric field to near objects that have a different potential level (especially the ground). The potential difference is essentially a difference in charge distribution. A typical example is that people sometimes feel mildly shocked when touching an appliance, even when the appliance is powered off. This is because there is a possibility of residual charge remaining inside the capacitors of the electronic circuits, which takes a little time to discharge. When the appliance is not appropriately grounded, touching it will cause a mild shock as the charge is transferred to the neutral body.

There are mainly two kinds of electric field-based HAR applications—active or passive—depending on the emitter of the field. An active electric field-based HAR application delivers the field variation as a signal source when the field is emitted from the environment and the human acts as an intruder. A passive one delivers the field variation when considering the electric field emitted from the body itself to the ground since the human body is a perfect conductor and can store the charges. The passive electric field describes a biological signal of the body, the human body capacitance, which will be introduced in the following subsection of the hybrid sensing technique in HAR. Here we firstly focused on the active electric field-based HAR application.

A very representative work is from Zhang et al. [116], where they introduced room-scale interactive and context-aware applications with a system named Wall++, which is a low-cost sensing technique that turns ordinary walls into smart infrastructures. The system can first track users’ touch and gestures and estimate body pose when close with the principle of active mutual capacitance sensing, which measures the capacitance between two electrodes (namely the electric field strength between the electrodes). When a body part is near a transmitter–receiver pair, it interferes with the projected electric field, reducing the received current, which can be measured for inferencing. On the other hand, if the user’s body touches an electrode, it dramatically increases the capacitance and the received current. Secondly, the system could also work in a passive airborne electromagnetic sensing mode to detect and track the active appliances and users when wearing an electromagnetic emitter. Another typical work is from Cheng et al. [117], where the authors used conductive textile-based electrodes that are easy to be integrated into garments to measure changes in the electric field strength (in capacitance) inside the human body. Since those changes are related to motions and shape changes of muscle, skin, and other tissue, the authors thus abstracted high-level knowledge from the changes and inferenced a broad range of activities and physiological parameters. For example, they embedded the prototype into a collar and performed quantitative evaluations of the recognition accuracy of actions such as chewing, swallowing, speaking, sighing (taking a deep breath), and different head motions and positions. There are other similar works based on active electric field sensing, such as touch detection [118], body tracking based on smart floor [119], respiration, heart rate, stereotyped motor behavior recording [120], hand gesture recognition [121], etc.

Active electric field sensing is non-intrusive, low-cost, has low power consumption, and has excellent potential for pervasive privacy-respecting environmental sensing. However, it is still more complex in hardware construction compared with the passive electric field sensing mode. Furthermore, it can be affected by electromagnetic interference. Thus its reliable operation has a demand in environmental conditions.

(B) Magnetic Field

Magnetic field sensing is an active approach for distance-based motion sensing. There are mainly two magnetic field-based motion-sensing systems depending on whether the magnetic field was generated by the direct current (DC) or alternative (AC) current.

In DC magnetic field motion sensing systems, electromagnets or permanent magnets are often used to generate the magnetic field. A magnetic sensor (magnetometer) senses the magnetic field strength. Since the magnetometer is widely embedded into wearable devices, the DC motion sensing system has been extensively explored for finger/hand tracking to enable a novel machine input approach. Chen et al. [122] designed a system named uTrack, which converts the thumb and fingers into a 3D input system using magnetic field sensing. A permanent magnet was affixed to the back of the thumb, and a pair of magnetometers were worn on the back of the fingers. A continuous data stream was obtained by moving the thumb across the fingers and was used for 3D pointing. The system shows a tracking accuracy of 4.84 mm in 3D space. Similar works [123,124] were conducted using a permanent magnet as the field generator for motion tracking.

In contrast, AC magnetic field sensing is mostly composed of oscillation-based magnetic field transmitters and receivers. The transmitter mostly uses coils to generate an alternating magnetic field. The receiver is also integrated with a coil to sense the strength of the magnetic field at different distances from the transmitter coil. This principle is that the oscillating magnetic flux through the receiver coils will induce an oscillating voltage with the same frequency. The voltage is later used for distance or pose estimation. Oscillating magnetic field has been explored in a variety of HAR tasks, such as indoor location [125], finger tracking [126], human–computer-interaction [127], wearable social distance monitoring [128,129,130], etc. It could also be implemented for underwater positioning to enable the tracking or navigation of underwater-unmanned vehicles or divers [131].

The advantage of the DC magnetic field motion sensing system is that the magnet used for field generating is easy to access. The sensing unit is at the chip level, thus enjoying the pervasiveness regarding the wide use of smart wearable devices. Moreover, the tracking accuracy can reach up to mm level. The disadvantage of such a system is located in the short sensing range. Since the field attenuates quickly, the detection range is limited to several centimeters. The AC magnetic field sensing’s performance in range and accuracy mainly depends on coil design. The detection range could reach up to ten meters with a larger transmitter coil. Ordinary everyday used furniture made of wood and textile will not deform the distribution of the activated field. However, the drawback is that the metallic objects will cause magnetic field distortions. Fortunately, researchers have tried to address this issue by a secondary calibration (either with a look-up table or with neural network-based calibration) step and achieved outstanding results [132].

(C) Gravitational Field

A gravitational field explains gravitational phenomena when a massive body produces a force on another massive body. Earth’s gravity is denoted by g, describing the net acceleration imparted to the physical objects caused by the combined effect of gravitation (caused by the mass distribution within Earth) and the centrifugal force (caused by Earth’s rotation). On Earth, gravity gives weight to physical objects. The weight is calculated by multiplying the gravitational acceleration by the mass. Gravitational field-based HAR tasks mainly utilize the pressure sensed by pressure sensors caused by the body’s weight. Different pressure sensors are presented for HAR tasks, such as the commercially available force-sensitive resistor (FSR), resistive textile, etc. By analyzing the pressure patterns caused by the motion of the body, extensive HAR applications are explored, such as gait analysis [133], workout recognition and user identification [134], indoor location [135], smart furniture [136], rehabilitation [137], etc. The textile-resistive pressure sensor is composed of a matrix of resistive units. By sensing the pressure of each unit from the matrix, the user motion patterns can be delivered. For a small number of resistive units, such as a few FSR units integrated into the insole, a one-dimensional data stream is used for action recognition. For a large number resistive units such as would be found on a mat-like surface, the data stream is usually converted to pressure images as two-dimensional arrays, which can be processed by a neural network-based algorithm used in computer version tasks for more accurate activity recognition.

One of the advantages of a pressure-based sensor is that the sensing component can be customized to any shape and size. Thus it is suitable for a large scale of surface types that needs to be sensed. The sensing precision could also be adjusted by arranging the density of the sensing units. Cheap, commercially available layer-wise films commonly construct the sensing unit. Thus the overall system is affordable to build. However, the cost comes into the system’s deployment in a large area (such as floors for location and tracking) since the sensing only occurs during contact, which is a drawback compared with other sensing modalities such as RF-based sensing with no limitation of contact. In summary, gravitational field-based HAR is a non-intrusive and straightforward motion action monitoring and analysis method. It can be extensively deployed for intelligent ambient sensing but is limited by the contact constraints and cost of deployment in a large area.

### 3.5. Hybrid / Others

(A) Human Body Capacitance

**Figure 10:**
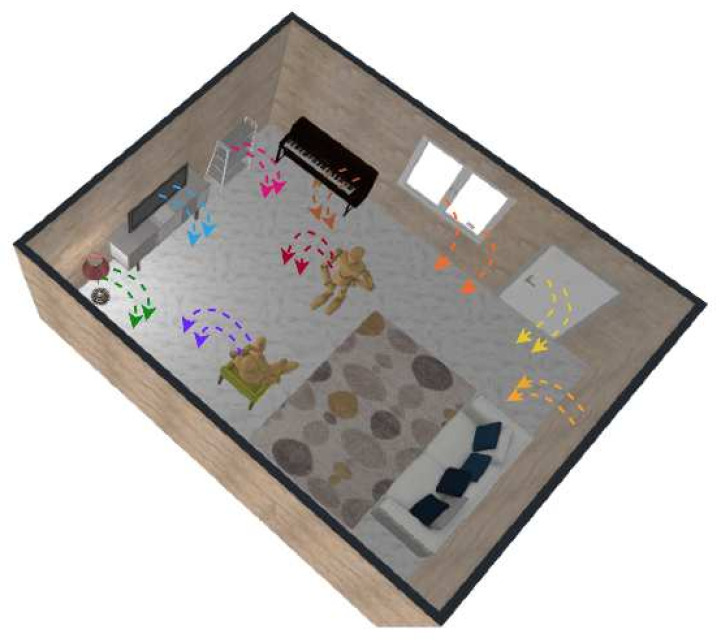
Human body capacitance: the static electric field between the body and the environment.

Human body capacitance (HBC) is essentially a biological variable describing the capacitance between the human body and the environment, mainly the ground. It is also a passive electric field-based sensing approach since the capacitance model comprises two conductive plates that store charges (corresponding to body and environment in the human electric field model) and a dielectric medium (corresponding to the air between body and ground). Figure 10 depicts the human body capacitance in a living room, where multiple electric fields exist, for example, the field between the appliance and the ground, between the metal frames of the window/door to the ground, as well as the human body capacitance between the body and the environment. person–person is a ubiquitous biological parameter that could be explored for a wide range of human-centric motion-related applications based on its sensitivity to both the body’s motion and the variation of the environment.

Unlike other biological features, such as ECG, EMG, etc., HBC is a feature that interacts with surroundings, especially the ground. Being insulated by the wearing, the body and the surroundings form a natural capacitor. HBC is used to describe the charges stored in the body. A series of studies [138,139,140,141] indicate a value of 100–400 pF of the body capacitance. The value varies with respect to skin state [142,143], garment [144], body postures [145], etc. Researchers have explored applications such as communication [146], cooperation perceiving [147], motion monitoring [117,148,149], etc., based on the concept, which has continued attracting the attention of researchers recently. Since HBC is a passive signal, the sensing units were mostly designed in a small form factor with small power consumption [149,150]. Wilmsdorff et al. [151] explored this passive capacitive sensing technique with a wide range of applications indoors and outdoors. In [152], the authors presented an HBC-based capacitive sensor for full-body gym exercise recognition and counting; by sensing the local potential variation of the body, different kinds of body actions could be classified. Besides motion sensing, HBC could also be used for proximity and joint activity recognition [147] by exploring the human body capacitance variation caused by the proximity and motion of an intruder.

As a passive motion-sensing approach, the systems based on human body capacitance enjoy the advantage of low cost, low power consumption, portability, and full-body sensing ability. However, although the sensitivity in motion and environmental variation forms the potential ability of this variable, at the same time they also limit the development of it, since any action, either from the body or from the environment, will induce an efficient signal, and there is difficulty in recognizing the source of the signal.

(B) Infrared

Infrared is electromagnetic radiation with wavelengths longer than visible light. The heat energy from the objects with a temperature above absolute zero is emitted as electromagnetic radiation, which is caused by the constant motion of molecules embodying heat. The electrons jump to higher energy band when they absorb energy by colliding with another. They can also release energy in the form of photons when falling to a lower energy band again. A hot molecule moves fast and generates higher frequencies (shorter wavelengths) of electromagnetic waves. Usually, the human eye cannot sense this radiation with infrared wavelengths, which can be measured by specific electronic sensors. Sensing the human body’s infrared could deliver information such as body temperature, motion trajectory, etc. Two kinds of sensors are commonly utilized for this purpose: the passive infrared sensor (PIR) and the thermographic camera.

The electronic sensor PIR is designed to measure infrared (IR) light from objects. The term passive indicates that this sensor does not emit energy during the detection process. Instead, it detects the energy of infrared radiation from objects. It is widely used from motion detection to automatic lighting applications. In the field of HAR, PIR has been widely explored in the application of indoor positioning [153,154], device-free activity recognition [155,156], etc. The sensor is widely available in the market with low cost and low power consumption. The built system is privacy-secure and easy to deploy and maintain. However, a PIR sensor only detects general movement. It does not give information on who or what moved. For that purpose, a thermographic camera for imaging IR is required.

A thermographic camera generates an image by infrared radiation, which is different from a common camera sensing visible light. The objects with a temperature above absolute zero can be detected by the thermographic camera, and an object with higher temperature emits more radiation. Thus from the thermography, the temperature variations are also visible. For example, humans and other warm-blooded animals stand out very well against the environment, regardless of whether it is day or night. Thermography has been widely used in medical diagnosis, in the military, etc. In HAR applications, it has also been developed with image-processing algorithms for activity detection in residential spaces [157,158], muscle activity evaluation [159], respiration monitoring [160,161], etc. For detection in dark lighting conditions, namely in the work performed by Uddin et al. [162], the authors used the OpenPose framework for thermal images to check the possibility of body skeleton extraction. Their result shows that the thermal images can monitor humans in dark environments where the other typical RGB cameras fail. Although thermographic sensing could supply more detailed information on body action than the PIR approach, it suffers lightly from the cost and the computing load.

### 3.6. Summary

Depending on the targeted application, researchers have explored different sensing modalities to accomplish their tasks in HAR. Table 2 summarized the mainstream of the sensing modalities and compared them with aspects of cost, power consumption level, working type (active or passive), privacy concern, computing load, typical applications, and their critical advantages and shortcomings. We also supplied some of the publicly available datasets of each sensing modality for HAR tasks in the table, so that readers can check and have a better understanding of the data properties of each sensing technique, or try their own mining approach on the dataset. The cost and power consumption express the practicability of a sensing modality, such as the IMU as a low-cost and low-power-consumption approach, which is the most widely explored aspect of HAR tasks. The compute load and robustness, ranging from high to low, were ranked with specific references. Computations that require large memory (over hundreds of megabytes) and complex instruction (such as multiplication of float point data) are regarded as having a high compute load. A low compute load needs simply a few instructions for one inference on weak devices such as the micro-controller. High robustness indicates that the signal could hardly be interfered with by surroundings, such as the gravitational field. Bluetooth signal strength, as an example, could be easily affected by a variation in the nearby environment. The typical application lists the activities coarsely at a high level, such as the activity of daily living (ADL), which includes all fundamental actions of a human in everyday life, such as sleeping, eating, dressing, etc. The positioning includes the location of the whole body and the body part such as the hand and finger. Gesture Recognition implies gestures performed by hand, finger, arm, etc. Active/passive sensors indicate the complexity of the sensing modality because of the existence of the signal sources. A privacy-respect sensor does not abstract identity-sensitive messages from users, thus being more acceptable. The computing load and robustness show the sensor’s working performance and are categorized into three levels: “low”, “medium”, and “high”. Depending on the usage scenarios, each sensing modality could be deployed targeting different tasks among “where”, “what”, and “how”. A passive electric field, as an example, can be used for both positioning and action sensing.

IMU sensor and optic approaches (mainly video-based) are the two most popular sensing modalities in the community, since the IMU sensor is pervasively deployed in smart devices and outperforms in power consumption, cost, size, and the visual modality can supply high accuracy for activity recognition benefiting from the advanced deep neural network models for feature abstraction. They both are utilized to target a much wider range of human activity recognition tasks than other sensors. However, there are still certain limitations, such as that they both suffer from computational load. Especially for the vision-based approach, which deals with 2D or 3D high resolution and high frame data stream with hundreds of thousands of conventional operations challenging the hardware resources, the computational load is high compared with other sensing modalities. Since the images from a video capture massive identity messages, the privacy issues need to be considered. The IMU sensors face accumulated errors, which results in the configuration demand for each new start for positioning applications with the demand of high accuracy.

Wave-based sensing modalities (RF waves and acoustic waves) are active approaches demanding signal sources from the sensing system and are mainly used for ambient intelligence. The corresponding systems are generally weak in robustness since the wave signal could be affected by the multipath effect (except for the UWB) and environmental noises. However, they are particularly efficient in privacy-respect scenarios since no other information beyond the wave property is collected. The cost and power consumption of such systems are much higher than the IMU-based solution, but still lower than the visual approach.

The electrophysiological signals (ExG) are perceived mostly by devices with high-resolution analog-to-digital chips for healthy monitoring such as mental state, stress level, sports quality evaluation, etc. The cost of such a system is relatively high compared with IMU and most field-based approaches. Since the signal sensing units are mainly at the chip-level design, the power consumption is an obvious advantage of those approaches. Depending on the channel numbers, the computational load of electrophysiological signals is distinct. The ECG signal, as an example, a simple rule-based approach that needs only a few computer instructions, can be used to detect the critical features from it efficiently. The EEG signal, on the other hand, requires a more complex algorithm to abstract the features from multiple channels to uncover the messages behind it.

Pressure sensing is versatile since the sensing unit (mainly composed of conductive layers) is highly customizable. Since the weight signal perceived from such a sensing system is quite straightforward, the detection accuracy of a certain human actions is high. However, maintaining such systems is costly because of the deployment complexity and the limited lifetime caused by the long-term stressful contact.

A magnetic field is a robust distance-based approach that can deliver reliable distance information with a lower computational load. The approach is low-cost and wearable (after minimizing), without limitation of multipath and line-of-sight. More importantly, it can be used for positioning in the underwater environment, which blocks most of the positioning techniques because of the quick attenuation of the adopted medium (such as RF-signal) in water. However, the detection range is limited by a few meters with the active magnetic field and a few decimeters with the passive magnetic field.

The electric field has recently become a novel sensing approach for HAR tasks, distinguished by its ability in full-body motion sensing and environmental electric sensing. It also enjoys the advantages of low power consumption and wearability. Since electrons exist anywhere in the environment where people live, including the human body, the body’s motion will deform the distribution of the electric field. Therefore, human activity could be deduced by perceiving the electric field variation, either on the environmental side or on the body side. However, the environmental noise is a big challenge for electric field-based sensing and is hard to overcome because of the pervasiveness of the surrounding objects acting as noise sources.

## 4. Outlooks

HAR relates to a wide range of tasks that deal with daily life with digitalization, aiming to assist people to have a better quality of life. As the keystone, sensing skills for HAR tasks are still under intensive development. Based on the surveyed most prominent sensing techniques in this manuscript, we further conclude some outlooks on the development of sensing skills for HAR tasks.

- **Sensor fusion**: The sensor fusion method has great potential to improve sensing robustness by fusing different sensor data. Each sensor modality has inherent strengths and weaknesses. By merging data from various sensor modalities, a robust data model can be generated. For example, the long-term positioning tasks with a high-rate IMU sensor will be disturbed by the integration errors, which could be addressed by a lower rate sensor that provides absolute anchor points (such as visual features). Some classic and efficient algorithms could be designed for sensor fusing, such as Kalman Filter [180]. As another example, the electric field sensors can perceive the straightforward proximity information of an individual. Meanwhile, they are sensitive to environmental variation, resulting in multi-source issues. By deploying motion sensors such as IMU on both individuals and the environment, the electric field signal source could be recognized. Such fusion approaches could not only address the weakness of a particular sensing modality but also provide a more holistic appreciation of the system being monitored.
- **Smart sensors**: Driven by the pervasive practical user scenarios and power-efficient data processing techniques, as well as the chip manufacturing technology, there is an apparent trend that sensors are becoming smarter with the ability to process the signal data locally. Compared to conventional sensor systems, smart sensors take advantage of emerging machine learning algorithms and modern computer hardware to create sophisticated, intelligent systems tailored to specific sensing applications. In recent years, many smart sensors have been proposed for HAR tasks such as the pedometer integrated IMUs (BMI270), gesture recognition integrated sensors (PAJ7620), etc. All the recognition, classification, and decision processes are executed on the smart sensor system locally instead of uploading the raw data to the cloud for inferencing. Thus, the user’s privacy is well protected, and the computing load of the central processing unit is significantly reduced.
- **Novel sensors**: With the development of materials and fabrication technology, novel sensing techniques and devices emerge to provide a broader perceiving ability towards the body and environment where people live. Novel sensors for HAR offer an alternative or complementary approach to existing solutions. More importantly, they supply a new method for body or environment knowledge collection that the current sensing technique cannot supply. An example is a microelectrode-based biosensor, which has been proposed for long-term monitoring of sweat glucose levels [181]. The multi-function microelectrode-based biosensor is fabricated on a flexible substrate, which offers greater wearing comfort than rigid sensors, thus providing long-term on-skin healthy monitoring.

Besides that, sensors are becoming more compact and power-efficient to provide always-on monitoring, or the sensors will be only in an active state triggered by a specific event. The energy harvesting techniques also provide ambient energy for sensors to extend the power life. With the growing number of wearable devices, the health monitoring sensors [182] are being deployed more near the body for continuous and real-time analysis of sweat, blood, etc., such as EEG monitoring by smart watches, or stress sensing by the Fitbit smart band, which uses an electrodermal activity skin response sensor to obtain a reading when the palm of user’s hand is pressing the metal outer rim, and then the corresponding app will analyze the overall stress.

## 5. Conclusions

This work focused on the mainstream sensing techniques for HAR tasks, aiming to supply a concrete understanding of the variant sensing principles for younger community researchers. We categorized the human activities into three classes: where, what, and how, for body position-related, body action-related, and body status-related services. This task-oriented categorization aims to supply a basic concept of the objectives of human activity recognition. We also categorized the HAR-related sensing modalities into five classes: mechanical kinematic sensing, field-based sensing, wave-based sensing, physiological sensing, and hybrid/others, based on the properties of the sensing medium, aiming to give a better understanding of the sensing technique’s physical background. Specific sensing modalities were presented in each category with state-of-the-art publications and a discussion of the modality’s advantages and limitations. A summary and an outlook of the sensing techniques were also discussed. We hope this survey can help newcomers have a better overview of the characteristics of each sensing modality for HAR tasks and choose the proper approaches for their specific applications.

## Figures and Tables

**Table 1:** Surveys on HAR sensing techniques.

Focused Subject	Ref	Year	Contribution
Device-free sensors	[4]	2020	Categorized sensors into wearable, object-tagged, device-free, etc. Focused on device-free sensing approaches for 10 kinds of activities. Extensive analysis based on 10 important metrics of each sensing approach.
Full-stack (sensors and algorithms)	[7]	2020	Categorized sensors into wearable, object, environmental, and video-based. Focused on data processing approaches.
Overall sensors	[8]	2020	Categorized sensors by physical principles (acoustic, optical, etc.). Summarized publicly available databases and common evaluation metrics to evaluate and compare the performance of the developed algorithms and systems.
Smartphone sensors	[5]	2019	Enumeration and description of embedded sensors. Data labeling, processing, etc.
Surveillance video	[9]	2019	Summarized the general process of human action recognition in video processing domain. Surveyed different features and models used in video surveillance, and the related datasets.
Radar sensors	[6]	2019	Overview of various radar systems adopted to recognize human activities. Overview of DL techniques applied to radar-based HAR tasks.
Bespoke sensors in smart home	[10]	2017	Highlighted that smart home intelligence involved sensing technology. Highlighted the multi-resident activity recognition including concurrent, interleave, and cooperative interaction activity.
Vision-based	[11]	2017	Comprehensive survey of different phases of vision-based HAR (image segmentation, feature extraction, activity classification).
WiFi-based	[12]	2016	Survey of the WiFi-based contactless HAR from four aspects including historical overview, theories and models, and key techniques for applications.
Non-invasive sensors	[13]	2016	Survey of technologies that are close to entering the commercial market or have only recently become available.
Vision-based	[14]	2015	Proposed categorization of human activities into unimodal and multimodal according to the nature of sensor data they employ. Reviewed various human activity recognition methods and analyzed the strengths and weaknesses of each category separately.
Wearable sensors	[15]	2014	Reviewed the latest reported systems on activity monitoring of humans based on wearable sensors. Forecasted the light-weight physiological sensors that lead to comfortable wearable devices to tackle the challenges.

**Table 2:** Sensing techniques in HAR tasks.

Modality	Cost (USD)	Power Level	Active/ Passive	Privacy	Compute Load	Robustness	Target	Typical Application	Comment	Accessible Dataset
WiFi	tens	≈tens Watt	active	no	medium	low	where, what	positioning, ADL, ambient intelligence	pervasiveness, environmental sensitivity	[163,164]
UWB	tens	≈mW	active	no	low	low	where, what	positioning, proximity, ADL, gesture recognition, ambient intelligence	multi-path resistive, high accuracy, costly for massive consumer usage	[165,166,167]
mmWave	tens	≈W	active	no	medium	low	where, what, how	positioning, proximity, ADL, gesture recognition, health monitoring, ambient intelligence	high accuracy, low power efficiency for massive consumer usage	[168,169]
Ultrasonic	hundreds	≈mW to W	active	no	low	low	where, what	positioning, proximity, ambient intelligence	high accuracy, weak robustness	-
Optic	tens of hundreds	≈W and above	passive	yes	high	medium	where, what, how	positioning, proximity, ADL, gait analysis, gesture recognition, surveillance	comprehensive approach, high resource consumption	[170,171,172]
ExG	hundreds	≈tens mW	passive	no	medium	high	how, what	sports, healthcare monitoring, ADL	high resolution, noise sensitive	[173]
IMU	a few	≈mW	passive	no	high	medium	where, what	positioning, ADL, gesture recognition, healthcare monitoring, gait analysis, sports	dominant sensing modality, accumulated bias	[174,175]
Magnetic Field(AC)	tens	≈hundreds mW	active	no	low	high	where, what	positioning, proximity,	high robustness, limited detection range	-
Magnetic Field(DC)	a few	≈mW	passive	no	low	high	what	proximity, gesture recognition	high accuracy, short detection range	[176]
Electric Field(active)	tens	≈mW	active	no	low	low	where, what	positioning, proximity, ambient intelligence	high sensitivity, noise sensitive	-
Electric Field(Passive)	a few	≈sub-mW	passive	no	low	low	where, what	positioning, proximity, sports, gait analysis, ambient intelligence	high sensitivity, noise sensitive	[177]
Gravitational Field	tens of hundreds	area dependent	passive	no	depends	high	where, what	positioning, sports, gait analysis, ambient intelligence	versatility/customizability, costly maintenance	[178,179]

## Data Availability

Not applicable.

## Abbreviations

The following abbreviations are used in this manuscript:

ToF	Time of Flight
RSS	Received Signal Strength
LiDAR	Light and Radar
UWB	Ultra-wideband
RF	Radio Frequency
ID	Identification
MAE	Mean Absolute Error
IMU	Inertial Measurement Unit
PWM	Pulse-Width Modulation
AUV	Autonomous Underwater Vehicles
SLAM	Simultaneous Localization and Mapping
